# The Student’s Manual of Histology

**Published:** 1882-12

**Authors:** 


### The Students' Manual of Histology.

For the use of students, practitioners and microscopists. Second edition. By Chas. M. Stowell, M.D., Assistant Professor of Histology and Microscopy, and the instructor in the Histological Laboratory of the University of Michigan. Illustrated by one hundred and ninety-two engravings. 290 pages.

Rapid advances are constantly being made in histological science, and it is highly important, for the sake of truth and correc-teaching, that works such as the above be issued often, at least sufficiently frequent, to have promptly presented all new discoveries, that truth may take the place of error. It is also highly important that all new and improved modes of investigation be clearly set forth, that students and investigators may pursue their work with the best facilities.

While the presentation of this branch of science in the larger works is of great value, they embrace much that, to say the least, requires revision. It is not possible that such works can keep abreast with the advances being made in this branch. It is only by the frequent issue of such works as the above that this can be done. This is a manual for the worker, and well does it fill the place.

Professor Stowell has been for years almost exclusively engaged in histological work, with special reference to lifting some subjects out of the realm of speculation and uncertainty, and placing them in the category of demonstrated truths ; and in addition to this exploring in new fields. He has had charge of, and been teaching in, the histological laboratory of the University of Michigan for the last four years, and indeed, has built up that department; so that his working experience enables him, well to understand the needs of the students, in respect to text books, and especially a manual from which to work. As a guide for the beginner this is equal if not superior to any work having a similar aim, hitherto issued.

The illustrations are very good. Some, of course, are copied from other works, but a large proportion are from original drawings by Prof. Stowell, from specimens now in his possession, each of which have been thoroughly proven. The work is published by George S. Davis, of Detroit, Mich., and can be obtained of booksellers generally.

